# Commentary: Comparison of Athletes' Proneness to Depressive Symptoms in Individual and Team Sports: Research on Psychological Mediators in Junior Elite Athletes

**DOI:** 10.3389/fpsyg.2016.01782

**Published:** 2016-11-18

**Authors:** Anne-Marie Elbe, Stine Nylandsted Jensen

**Affiliations:** Department of Nutrition, Exercise and Sports, University of CopenhagenCopenhagen, Denmark

**Keywords:** maladaptive perfectionism, attributional style, cohesion, mental health, depression

Depression, one of the most commonly diagnosed mental health disorders, is an emerging public health problem (Andersen et al., 2011). Depression is defined as frequently experienced depressive moods, loss of interest or pleasure, decreased energy, feelings of guilt or low self-worth, disturbed sleep or appetite, and poor concentration (American Psychiatric Association, 2013). As a consequence, depression increases physical distress and health problems, ultimately impairing functional well-being and quality of life (American Psychiatric Association, 2013). It has been estimated that approximately 15% of the population worldwide is at risk of developing depressive symptoms (Richards, 2011; Vilhelmsson, 2013). While research on depression in the general public is extensive, research on depression in the sport context, however, is limited. The few studies that do exist, outlined in Nixdorf et al.'s (2016) introduction, identified prevalence rates of 4–68%. A main research focus has been on sport's antidepressant function and how regular physical activity can reduce depressive symptoms, preventing the occurrence of depression (Babiss and Gangwisch, 2009). In line with this research it has been assumed that because elite athletes are so physically active they are immune to depression. Recent studies (Hammond et al., 2013; Gulliver et al., 2015) have, however, highlighted that elite athletes might be just as likely as non-athletes to experience depression, and that psychosocial benefits attributed to sport do not inherently occur through mere sport participation. Studies in the general population indicate that young people are especially at risk for depression due to the developmental challenges and life transitions they face (Suvisaari et al., 2009; Gulliver et al., 2015). Because junior elite athletes, who face the same developmental challenges as all other young people, are additionally confronted with challenges inherent in elite sports, research on depression in this specific population seems especially warranted.

While strides have been made in the classification, assessment, and identification of depression, given its complexity, the cause of depression is difficult to articulate, and has not been adequately elucidated (Beck and Alford, 2009). It is therefore particularly important to examine which sources may contribute to depression among elite athletes as these might differ from those affecting non-elite athletes. Nixdorf et al. (2016) nicely outlined the risks related to being an elite athlete (e.g., injuries, overtraining, or simply competing in a different sport discipline) and illustrated well how these risks might be related to developing depression. Nevertheless, there is a noticeable research gap when it comes to investigating personality and social factors as possible precursors of depressive symptoms among junior as well as senior elite athletes. Therefore, examining attributional style, perfectionism, and cohesion in relation to depressive symptoms seems highly relevant.

Nixdorf et al.'s (2016) finding that attributional style after failure mediated depression in individual sport athletes is important for several reasons. Attributional style, which refers to the reasons individuals give for their success and failure, has been shown to be related to emotional well-being (Allen, 2012). Not only can this finding help explain how depression develops; it can also inform recommendations for practical interventions. Attributional styles can be changed through systematic sport psychological training in such a way that they enhance positive emotions (Beckmann and Elbe, 2015). Hence, if athletes were taught how to talk to themselves better after they have experienced failure, this might contribute to alleviating depressive symptoms and at the same time also enhance motivation. It has been shown that attributing failure to external, unstable, and specific factors is a more adaptive attributional style than blaming failures on internal, stable, and global factors (Buchanan and Seligman, 1995).

It is also interesting that maladaptive perfectionism and low cohesion were related to depression in Nixdorf et al.'s (2016) study. The result concerning maladaptive perfectionism is in line with other studies suggesting a relationship between maladaptive perfectionism and depression (Ashby et al., 2012; Zhou et al., 2013; Noble et al., 2014). The self-blame/defeating component related to maladaptive perfectionism, which is similar in nature to an attributional style, that blames failure on internal, stable, and global factors could be the decisive factor for the occurrence of depression.

Last but not least, the paper clearly identifies that young elite athletes participating in an individual sport are at a higher risk for depressive symptoms than team sport athletes. This finding is consistent with previous research examining the health advantages of team sports over individual sports. The studies investigating different types of physical activity interventions and which indicated that team sports are more conducive for participants' motivation (Nielsen et al., 2014), experiences of flow (Elbe et al., 2010), and cardiovascular health (Krustrup et al., 2010), could possibly explain why young elite athletes participating in individual sports are more at risk for depressive symptoms than older athletes.

This paper addresses an important issue because psychological well-being and mental disorders have increasingly become of public and scientific interest in elite sports, particularly since several cases of prominent elite athletes affected by depression have become publicly known (Nixdorf et al., 2013). The European Federation of Sport Psychology (FEPSAC) is currently working on the topic, developing a position statement related to the mental health of elite athletes.

Knowledge of psychological and social factors related to depression in elite athletes is scarce. This paper, therefore makes an important contribution to the knowledge about the mental health of young athletes but also gives a useful recommendation for applied practitioners, namely, to pay attention to athletes' attributional styles. This study illustrates that the mental health of young athletes needs stronger attention in order to prevent depressive symptoms and depression, especially in individual sport athletes. Given the fact that specific challenges are associated with depression like the comorbidity with other mental disorders such as anxiety and eating disorders, it is important to keep the mental health of young athletes in focus. Due to the stigma related to depression and the fact that athletes might hesitate to seek help, providing them with information about how and where to seek help should be a priority. Individuals in contact with young athletes like coaches, parents, and physical therapists should be informed about support services for young athletes with depressive symptoms so that they can be referred to them. Finally, longitudinal studies are needed to better understand the duration of depressive symptoms in young athletes and causal relationships between depression and psychological precursors.

## Author contributions

AE and SN contributed equally to the writing of this commentary.

### Conflict of interest statement

The authors declare that the research was conducted in the absence of any commercial or financial relationships that could be construed as a potential conflict of interest.
